# Cost for sickle cell disease screening using isoelectric focusing with dried blood spot samples and estimation of price thresholds for a point-of-care test in Uganda

**DOI:** 10.2147/JBM.S186528

**Published:** 2019-02-05

**Authors:** Mercy Mvundura, Charles Kiyaga, Mutsumi Metzler, Carol Kamya, Jeanette M Lim, Catherine Maiteki-Sebuguzi, Patricia S Coffey

**Affiliations:** 1 Devices and Tools Global Program, PATH, Seattle, WA, USA, pcoffey@path.org; 2 Uganda Central Public Health Laboratory, Ministry of Health, Kampala, Uganda; 3 Evaluation Projects, Infectious Diseases Research Collaboration, Kampala, Uganda; 4 Independent Consultant, Kampala, Uganda

**Keywords:** point-of-care, sickle cell disease, costing, Uganda, diagnostics, screening

## Abstract

**Background:**

Early identification through newborn screening is the first step in active management of sickle cell disease (SCD). Uganda currently screens newborns and infants under 2 years for SCD in high HIV-burden districts using isoelectric focusing with dried blood spot samples. Our analysis sought to estimate the costs per child screened for SCD using this method in Uganda and then to use those data to estimate the price threshold for screening with a point-of-care (POC) test.

**Methods:**

We estimated the financial and economic costs per child screened for SCD using data from health facilities and the Central Public Health Laboratory. These costs included sample collection, transportation, and laboratory processing. Price thresholds for a POC test were estimated using two scenarios.

**Results:**

The price threshold of an SCD POC test used for diagnosis would be $3.77 when taking into account only financial costs and $5.14 when taking into account economic costs. Thresholds for a POC test used for screening would be $3.07–$3.51 and $4.38–$5.09, respectively, depending on test specificity.

**Conclusion:**

The price threshold of a POC test for SCD will depend on the assumptions on how it will be used – either as a screening or diagnostic test. If used for screening, test specificity will have significant impact. Results from this type of costing study can allow developers to incorporate quantitatively estimated price thresholds for innovative products into target product profiles early in the product development cycle.

## Background

Sickle cell disease (SCD) is an aggregate of several genetic blood disorders that involve the presence of a sickle cell gene. In SCD, at least one of the beta-globin subunits in hemoglobin is replaced with hemoglobin S. In sickle cell anemia, which is a common form of SCD, hemoglobin S replaces both beta-globin subunits in the hemoglobin.^1^ SCD is one of the most common single-gene disorders worldwide. The disease causes a debilitating systemic syndrome that includes chronic anemia, acute painful episodes, chronic organ damage, and a significant reduction in life expectancy. About 80% of the affected children are born in developing countries, three-quarters of whom are in low- and middle-income countries (LMIC) of sub-Saharan Africa.^2^ SCD accounts for the equivalent of 5% of under-five deaths in Africa, with an estimated 50%–80% of children born with SCD dying before age 5.^3^ Early identification of SCD through newborn screening is the first step in active management of this disease condition.^4^ In 2008, the United Nations General Assembly recognized sickle cell anemia as a public health problem.^5^ In 2010, the WHO defined an SCD strategy for the WHO African Region, which included screening of newborns.^6^

A few LMIC have initiated SCD screening programs, including Uganda where the prevalence of children with SCD was estimated to be 0.73% and the prevalence of children with sickle cell trait was estimated to be 13.3% in 2015.^7^ To address this, Uganda introduced newborn SCD screening into an existing program that identifies and treats HIV-infected mothers and their exposed infants in high-HIV burden districts. In this SCD program, dried blood spot (DBS) samples are collected from newborns at health facilities after birth and before discharge and from children under 2 years at health facilities during immunization visits and/or during inpatient or outpatient visits. Samples are sent to the Central Public Health Laboratory (CPHL) for analysis using isoelectric focusing (IEF). The SCD newborn screening program leverages the sample transport network used for transporting DBS samples for the early infant diagnosis (EID) program. This screening system relies on a good logistical system for sample transportation to and from the CPHL and also requires a functional and financed system for relaying results to caregivers. For remote facilities, the logistical challenge of transporting samples to and results from the CPHL can negatively impact the screening program. In addition, without a well-resourced system for relaying results to caregivers, loss to follow-up could be high and hence delayed treatment may be another challenge for the program.

Newborn screening with a point-of-care (POC) test that is performed at primary health facilities with results given to caregivers during the same visit would allow earlier detection, education, and management to more effectively prevent early mortality in infants and children with SCD.^8^ Current efforts to address this technology gap by developing POC tests for low-resource settings are underway. Some SCD POC tests have already been commercialized. Sickle SCAN^®^ (BioMedomics, Inc., Research Triangle Park, Durham, NC, USA), a multiplexed qualitative POC immunoassay used for the rapid diagnosis of SCD has recently been commercialized at a price point of around $4.50 per test.^9^ This test has shown promising results including laboratory tests on 139 samples using venous blood, which demonstrated high sensitivity and specificity for the detection of HbA, HbS, and HbC.^10^ HemeChip (Hemex Health, Portland, OR, USA) is a disposable test chip and miniature reader that tests blood using cellulose acetate electrophoresis.^11^,^12^

These POC tests are, however, relatively unavailable in LMIC currently and the question remains regarding what the target price of an SCD POC test should be in order for the test to be affordable to public sector programs in LMIC. The target price is one of the characteristics in a target product profile (TPP), which technology developers should develop in the early stages of their product development process in order to ensure alignment between the performance and operational characteristics of the products that they develop and the needs of end users. Unfortunately, this important aspect of early product development does not often take place with the appropriate rigor. Qualitative analyses, such as interviews and discussions with experts, have often been employed to determine the target price for a product used in low-resource settings. Also, potential buyers’ past spending on similar products and competitive products that are on the market are also sometimes taken into account.^13^ However, in order to estimate the target price for a product, it is important to consider not only the market price but also the economic costs to implement the new technology in a programmatic setting. These economic costs include both financial costs (such as cost of the test and supplies) and also the opportunity costs of using resources associated with service delivery. It is also important to benchmark these costs with the costs of existing tests, as this helps inform the comparative value of the new test vs existing tests. Data generated from costing studies can help fill this gap.

Costing analyses of SCD newborn screening in LMIC are sparse. One study of a newborn screening program in Angola that used DBS for screening estimated that the cost per infant screened was $15.36 in 2011 international dollars.^14^ Another study estimated that the costs for screening using DBS in Uganda would be $9.94 but it is unclear how these costs were estimated.^15^ None of these studies compared the costs of POC tests to DBSs processed using IEF. Therefore, in order to better inform the target price of an SCD POC test, we conducted a costing study to estimate the financial and economic cost per child screened and diagnosed for SCD under a current program in Uganda. Financial costs represent actual expenditures for goods and services; these costs are usually included in the budgeting process. Opportunity costs capture the cost of foregone use of existing resources; eg, human resource time for staff who are already employed by the government. Economic costs are the sum of the financial and opportunity costs. We used the data from the costing study to estimate the price thresholds for an SCD POC test such that a program using an SCD POC test would have comparable costs to the current program, which uses IEF with DBS.

## Methods

### Study site selection

We selected Uganda for this costing study because it has been implementing a newborn screening program for SCD, and therefore it has a program in place where we could obtain data to estimate economic cost per child screened. Six health centers (five level III health centers and one level IV health center) were selected for inclusion in the costing study, as these were the ones performing SCD screening. These health facilities were providing newborn screening for SCD in urban and peri-urban areas in and around Kampala.

**Figure 1:**
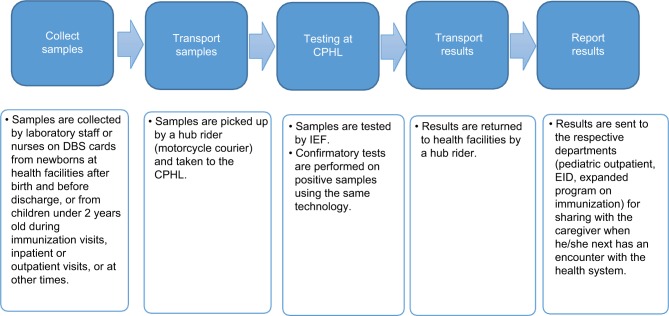
Process for sample selection until relay of results. **Abbreviations:** CPHL, Central Public Health Laboratory; DBS, dried blood spot; EID, early infant diagnosis; IEF, isoelectric focusing.

As mentioned before, Uganda is using IEF with DBS for SCD screening and diagnosis. IEF requires laboratory equipment, infrastructure, and technical ability to implement successfully. Electrophoresis equipment or automated systems require electricity, and consumables in the form of reagents and gels require refrigeration. Figure 1 explains the process employed from sample collection to relay of results to caregivers in the health facilities included in this costing study. Samples are collected on DBS cards, which are then sent to the CPHL by motorcycle courier. The CPHL then tests those samples using IEF. If samples tested positive, confirmatory tests are performed using the same technology and then test results are sent back to health facilities.

### Data collection methods

Structured questionnaires were used to collect data from staff at six health facilities and from the CPHL, concerning the quantity and unit cost of resources used in screening and diagnosing SCD. Either one nurse or one laboratory staff responsible for specimen collection was interviewed at each of the six health centers to provide information on the resources used to collect DBS from a typical child. One laboratory staff at the CPHL was also interviewed to provide information on supplies, reagents, instruments, and equipment used to process the DBS and human resource time for each laboratory process. The CPHL staff also provided information on batch processing and capacity in the laboratory to enable estimation of costs per DBS sample processed. All interviews were conducted in October 2016. Study staff obtained verbal consent from study participants prior to initiating the interviews. Study participants did not receive any compensation for their involvement in the costing interview, which took about 45 minutes to complete.

### Costs for resources included in the analysis and assumptions

The costs for the resources included in this study were 1) costs of supplies used for sample collection (the DBS kit) at the health facility and gloves; 2) costs of health facility staff time spent on counseling the caregiver about SCD and on sample collection and packaging; and 3) sample transportation costs. The DBS kit contained a filter paper card, alcohol swab, lancet, and capillary tube in a plastic bag with a desiccant. Costs for the motorcycle couriers used for sample transportation included the annualized purchase costs of the motorcycles, fuel, service and maintenance, and the courier/motorcycle driver allowance. The couriers transported DBS samples for both the EID and the SCD screening programs and also other specimens. The cost per sample or specimen was estimated by dividing the courier costs by the total number of samples and specimens transported per year.

At the CPHL, the cost included the costs of supplies, reagents, instruments, and equipment used to process the DBS cards and staff time spent on sample processing. The CPHL staff reported that they processed 80 specimens per batch and ~200–300 specimens per day. We used these numbers to estimate cost of sample processing.

Both financial and opportunity costs were collected and included in this study. As mentioned above, financial costs represent actual expenditures for goods and services and these costs are usually included in the budgeting process. Opportunity costs capture the cost of foregone use of existing resources, eg, human resource time for staff who are already employed by the government. Economic costs are the sum of the financial and opportunity costs. We included both financial and opportunity costs in our estimates because the financial costs are the ones that have budgetary implications, but it is also important to consider the opportunity costs (and hence the full economic costs) as this will show the economic burden to the program.

Table 1 shows the classification of economic costs estimated in this study as either financial or opportunity costs. We did not distinguish between the sources of the funding for the resources. Hence, the study includes the costs of resources funded by the ministry of health as well as resources provided through donations.

We made certain assumptions when estimating the costs for the current program or when estimating the costs for a program using an SCD POC test. These assumptions include: 1) each staff works 20 days a month and 8 hours a day; 2) the courier serves 25 health facilities each month and collects samples for various tests, including the DBS card samples for the SCD and EID programs and other specimens; 3) health workers spend the same amount of time providing SCD counseling to caregivers regardless of whether the current program or a program using an SCD POC test is utilized; 4) collecting a sample and running an SCD POC test takes 25 minutes; and 5) an SCD POC comes as a full kit containing a lancet, capillary tube, and alcohol swab, and therefore health facilities additionally require only gloves to conduct an SCD POC test, just as they now do for the current program.

Unit costs of resources that were locally procured were obtained from the Joint Medical Store price list. Unit costs for resources that were imported (eg, all donated and imported reagents and equipment used at the CPHL) were obtained from the Internet and verified through a price quotation from the main supplier. Table 2 shows costs of major resources used to estimate the economic costs.

### Data analysis

The analysis was done using Microsoft Excel (Redmond, WA, USA). All costs were reported in 2016 US dollars and an exchange rate of 3,458 Ugandan shillings per US$1 was used for currency conversion. We first estimated the financial and economic costs per child diagnosed under the current SCD screening program by summing the costs of all the resources used. We then used these estimates to calculate the price thresholds (the possible price) of an SCD POC test in order for the financial and economic costs per child screened and diagnosed to be comparable between the current program and a program using an SCD POC. These calculations were done using the Excel Goal Seek function.

In order to estimate the price thresholds, we considered two scenarios of an SCD POC test: 1) use as a screening test and 2) use as a diagnostic test. “Screening” refers to a single use (one DBS sample or POC test), and “diagnosis” refers to conducting the test a second time (a second DBS sample or POC test) to confirm the screening test result. In the screening use case, positive samples would be collected on DBS cards and tested with IEF at the CPHL. We assumed that the sensitivity of the SCD POC test would be 99%, similar to Sickle SCAN, and varied the specificity at 99%, 95%, 90%, and 85% to explore its impact on the estimated price thresholds. Based on Ndeezi et al (2016)^7^, we assumed that 14% of children had SCD or its traits.

### Ethical considerations

This study protocol and verbal consent process was approved by the Mulago Research Ethics Committee at Mulago National Referral Hospital in Kampala and the Uganda National Council for Science and Technology. Verbal consent was received from study participants prior to initiating the interviews.

## Results

The staff at health facilities reported that they spent an average of 7.5 minutes (range 1.5–15 minutes) providing SCD counseling to caregivers before sample collection, an average of 4 minutes (range 2–5 minutes) collecting and packaging a DBS sample, and an average of 6.75 minutes (range 3.5–7.5 minutes) communicating SCD screening results to caregivers. The total cost of staff time at health facilities per child screened for SCD was estimated at $0.24 (range $0.14–$0.38), as shown in Table 3. Including costs of staff time, sample collection supplies, and sample transportation, the average total costs at health facilities per child screened for SCD using DBS were estimated to be US$1.74 (range $1.59–$1.97).

At the CPHL, cost per SCD sample processed was estimated at $4.14 including the time of the laboratory staff and the cost of lab suppliers including reagents. As a result, the average economic cost per child screened for SCD with the current method of DBS was estimated to be US$5.88 on average (range $5.73–$6.11) of which $3.67 were financial costs (Table 3).

If an SCD POC test was to be used as a diagnostic test, the result of an SCD POC at a health center will become the final result. Only additional item required to conduct an SCD POC test is a pair of gloves ($0.13) used for sample collection because these are not included in the POC kit. As Table 4 shows, the financial price threshold for an SCD POC would be $3.54, and this estimate is calculated as the difference between the average financial costs used under the current program and the financial costs not included in the SCD POC test ($3.67–$0.13). This is the price threshold to use if we account only for the costs of resources that would have direct budgetary implications and exclude the opportunity costs of using existing resources. If we consider the full economic cost, we will need to take into account the opportunity costs. The costs for staff time at health facilities was estimated to be $0.61, assuming that health facility staff would spend the same amount of time on counseling and relaying results to the caregivers as in the current system but would spend 25 minutes on running the SCD POC test. The cost for sample transport to and processing at the CPHL would be eliminated if the POC were to be used as a diagnostic test. Therefore, in order for a program using an SCD POC test to be comparable in economic cost to the current program, the price threshold of the SCD POC test would be $5.14 – the difference between $5.89 and $0.74 ($0.13 for gloves + $0.61 for staff time).

**Figure 2:**
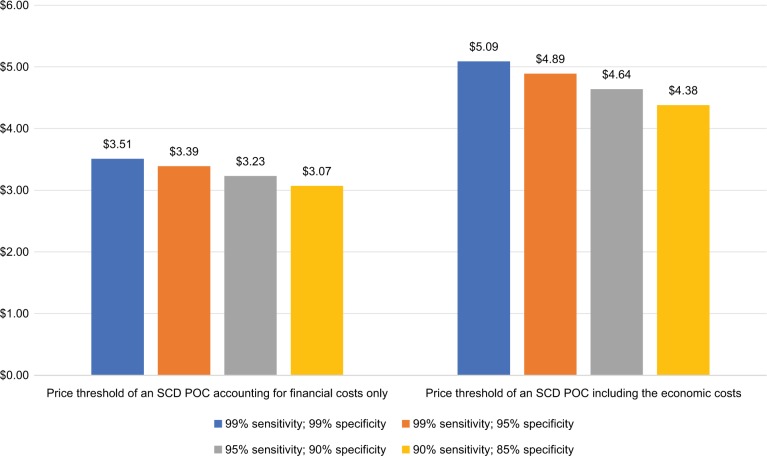
Financial and economic price thresholds for an SCD POC test that is used for screening. **Abbreviations:** POC, point-of-care; SCD, sickle cell disease.

If an SCD POC test is used as a screening test, costs associated with conducting an additional test will need to be taken into account. In this case, the specificity of an SCD POC affects the number of children who will be sent to receive an additional test and thus the costs. We therefore examined the effects of the test specificity by varying it while holding sensitivity constant. When only accounting for the financial costs of the current program, we estimated that the price thresholds for an SCD POC ranges from $3.07 to $3.51 depending on the specificity of the SCD POC test (Figure 2). We estimated that the price threshold for an SCD POC would range between $4.38 and $5.09 to make it comparable to the economic costs of the current program, and the price threshold also depends on the specificity of the SCD POC test (Figure 2).

The price threshold, or the maximum price, for an SCD POC test used for screening will always be lower than the price of an SCD POC used for diagnosis. This is because a certain number of children are always referred for a diagnostic test, incurring the cost of performing the tests, when an SCD POC is used as a screening test. When the specificity of the test is low, there will be many false positive test results from the screening test and this increases the number of children unnecessarily referred for an additional diagnostic test.

## Discussion

Affordable POC tests to identify SCD have been hailed as an appropriate solution for LMIC.^16^ Our cost study showed that the price threshold of a SCD POC test would be $3.77 when taking into account only financial costs and $5.14 when taking into account economic costs, if the test is used as a diagnostic test. If it is used as a screening test, these thresholds will be $3.07–$3.51 and $4.38–$5.09, respectively, depending on the specificity of the SCD POC test.

Based on the Internet prices, we noted that some of the SCD POC tests that are available or will be on the market are below the financial price threshold while some are above. POC tests currently on the market range in price from $2.30 to $9.40 per test. However, even for the SCD POC tests with Internet prices that are below the financial price thresholds we estimated, it should be noted that the landed prices for these POC tests will be higher than the listed Internet prices because shipping, customs clearance, and other in-country costs such as regulatory approval costs are not included in these online prices. Also, these online prices do not account for other supplies that are required to run these tests that are not bundled with these tests. Therefore, the online prices cannot be directly comparable with the financial price threshold estimates we generated. Also, the sensitivity of these tests to detect SCD are generally close to 100%, while specificity ranges from 90.9% with SICKLEDEX to 90%–100% for Sickle SCAN and HemeChip.^17^–^19^ The results of our analysis will be informative for developers of tests that have lower specificity.

We included both financial and opportunity costs in our price threshold calculations. While financial costs are the ones that are likely to have budgetary implications, it is important to consider the opportunity costs and hence the economic costs. Accounting for the opportunity costs helps to shed light on the economic burden of a test. For example, a test may be offered at a lower price but it can place a strain on existing resources by, eg, being time-consuming for health care workers to run. If only financial costs are accounted for, this type of burden on existing resources will be overlooked. Also, cost-effectiveness analyses include economic costs and hence the estimates generated will be informative for future studies that will compare the cost-effectiveness of different tests for SCD.

There are some limitations to this cost study. First, estimation of the economic costs was based on data from a small sample of facilities that were located near Kampala, so these few sites may not be representative of the system in the whole country. Our costs may underestimate the economic costs, especially for transport, which may be higher for facilities that are farther away from the CPHL, which is located in Kampala. Also, our estimated sample transport costs are low because the SCD DBS samples are transported leveraging the transport system of the HIV EID and thus creating efficiencies. In other countries, where there are no existing sample transport systems to leverage, having a test that requires transport to a centralized location for processing may result in much higher sample transportation costs. Second, the cost estimates do not include several costs, such as shipping and handling charges, customs clearance for imported supplies and equipment, equipment maintenance and repair costs, supervision and management costs, and treatment costs, and hence may again underestimate the costs for the current program. In addition, the estimated price thresholds of the POC tests may be overestimated because we did not factor in the training of users to conduct the POC tests and also quality assurance and supervision. Finally, the study does not include costs borne by caregivers or their households such as transport costs to bring children to health facilities and the opportunity costs of caregiver time. If all these costs are included, the economic costs of the current program and a program utilizing an SCD POC would likely increase, and that would affect the price threshold estimates of an SCD POC test. Importantly, our analysis assumes that the two test approaches demonstrate similar levels of sensitivity and specificity and are able to detect the same hemoglobins.

Technology developers frequently develop TPPs, including the target product price, in order to ensure the product that they are developing is well aligned with the needs of the users at an acceptable price. However, for developing countries, some of these TPPs are not informed by evidence from the countries where these products are intended to be used. As a result, sometimes products’ features do not fit the needs of LMIC and/or prices are beyond what the public health systems in developing countries can accept. Although our cost study has some limitations, it quantitatively shows the price thresholds for an SCD POC test. We hope this result can assist test developers and manufacturers to determine the appropriate target price for an SCD POC test from both a financial and an economic perspective. Our cost study has generated estimates that may be useful in the early stages of product development to assess one aspect of product marketability and to determine if a POC test warrants further investment for development, validation, and commercialization.

## Conclusion

The price threshold of a POC test for SCD will depend on the assumptions on how it will be used – either as a screening or diagnostic test. If used as a screening test, test specificity will have significant impact. Results from this type of costing study can allow developers to incorporate quantitatively estimated price thresholds for innovative products into TPPs early in the product development cycle.

## Data sharing statement

The dataset generated and analyzed during the current study is available from the corresponding author on reasonable request.

## Figures and Tables

**Table 1:** Classification of economic costs included in the analysis

Economic costs included in the analysis	Financial costs	Opportunity costs
Staff time		X
Sample transportation^a^		X
Supplies for sample collection	X	
Instrument and equipment at CPHL		X
Consumables (reagents and supplies) at CPHL	X	

**Note:**

a Sample transportation was classified as an opportunity cost because this was a shared resource with the HIV EID program and other programs, so even if the SCD program was not using the sample transport system, these costs would continue to be incurred by the government.

**Abbreviations:** CPHL, Central Public Health Laboratory; EID, early infant diagnosis; SCD, sickle cell disease.

**Table 2:** Key costs for major resources used

Inputs	Amount (USD)
Monthly salaries	
Nurse at a health facility	$119
Laboratory technician at a health facility	$180
Laboratory staff at the CPHL	$600–$1,200
Supplies for DBS sample selection	
Filter paper card	$0.73
Alcohol swab	$0.04
Lancet	$0.05
Capillary tube	$0.16
Storage bag	$0.15
Desiccant	$0.30
Gloves	$0.07
Courier costs	
Monthly allowance	$200
Cost of a motorcycle	$3,800
Selected supplies used in the CPHL	
PerkinElmer resolve system	$3,304
Stains	$293
Reagents	$484
Selected equipment used in the CPHL	
Puncher	$18,000
Electrophoresis supply system	$7,500
Water bath	$7,000
Shaker	$2,000
Dryer	$1,100

**Abbreviations:** CPHL, Central Public Health Laboratory; DBS, dried blood spot.

**Table 3:** Economic and financial costs for a child/sample screened and diagnosed under the current program with DBS processed using IEF

Cost category	Economic costs per child in current program: average (range), (USD)	Financial costs per child in current program: average (range), (USD)
Health staff time to counsel caregiver	$0.10 ($0.02–$0.20)	NA
Health staff time to collect samples and packing	$0.05 ($0.03–$0.09)	NA
Health staff time to share results to caregiver	$0.09 ($0.05–$0.10)	NA
Subtotal cost for staff time at health facilities	$0.24 ($0.14–$0.38)	NA
Sample transportation	$0.03	NA
Supplies for sample collection at health facilities	$1.47 ($1.42–$1.56)	$1.47 ($1.42–$1.56)
Total cost per child at the health facility	$1.74 ($1.59–$1.97)	$1.47 ($1.42–$1.56)
Laboratory technician time to run test at CPHL	$1.24	NA
Instrument and equipment at CPHL	$0.70	NA
Consumables (reagents and supplies) at CPHL	$2.20	$2.20
Total cost per child at CPHL	$4.14	$2.20
Total cost per child screened and diagnosed	$5.88 ($5.73–$6.11)	$3.67 ($3.62–$3.76)

**Abbreviations:** CPHL, Central Public Health Laboratory; DBS, dried blood spot; IEF, isoelectric focusing; NA, not applicable.

**Table 4:** Price threshold for a diagnostic POC test that would make the financial and economic costs similar to the current program

Cost category	Current program average costs	POC as diagnostic test
**Financial costs**		
Supplies at health facilities	$1.47	$0.13
Reagents and supplies at CPHL	$2.20	0
Total financial costs per child in current program	$3.67	
Price threshold for POC test that would make the financial costs for a program using an SCD POC similar to that of the current program		$3.54
Subtotal financial costs per child screened	$3.67	$3.67
**Opportunity costs**		
Staff time at health facilities	$0.24	$0.61
Sample transportation	$0.03	0
Staff time at CPHL	$1.24	0
Instruments and equipment at CPHL	$0.70	0
Total economic costs (financial plus opportunity costs) per child in current program	$5.88	
Price threshold for POC test that would make the economic costs for a program using an SCD POC test similar to the current program		$5.14
Total economic costs per child screened	$5.88	$5.88

**Abbreviations:** CPHL, Central Public Health Laboratory; POC, point-of-care; SCD, sickle cell disease.
